# Long non-coding RNA MEG3 promotes fibrosis and inflammatory response in diabetic nephropathy via miR-181a/Egr-1/TLR4 axis

**DOI:** 10.18632/aging.102011

**Published:** 2019-06-13

**Authors:** Fangfang Zha, Xiaolu Qu, Bo Tang, Ji Li, Yakun Wang, PengXi Zheng, Tingting Ji, Chun Zhu, Shoujun Bai

**Affiliations:** 1 Department of Nephrology, Qingpu Branch of Zhongshan Hospital Affiliated to Fudan University, Qingpu District, Shanghai 201700, P.R. China; 2 Department of Nephrology, Shanghai Punan Hospital of Pudong New District, Pudong New District, Shanghai 200215, P.R. China; 3 Department of Nephrology, Xinhua Hospital Affiliated to Shanghai Jiao Tong University School of Medicine, Chongming Branch, Chongming District, Shanghai 202150, P.R. China; 4 Department of Nephrology, Xinhua Hospital, School of Medicine, Shanghai Jiao Tong University, Yangpu District, Shanghai 200092, P.R. China

**Keywords:** diabetic nephropathy, lncRNA MEG3, miR-181a, ceRNA

## Abstract

Long non-coding RNAs (lncRNAs) play vital roles in diabetic nephropathy (DN). This research aimed to study the potential role and underlying molecular mechanisms of long non-coding RNA MEG3 in DN. We found that MEG3 was upregulated in DN in vivo and in vitro and could enhance cell fibrosis and inflammatory response in DN. MEG3 functioned as an endogenous sponge for miR-181a in mesangial cells (MCs) via direct targeting and in an Ago2-dependent manner. MiR-181a inhibition promoted MC fibrosis and inflammatory response. In addition, Egr-1 was confirmed as a target gene of miR-181a. Further investigations verified that MEG3 promotes fibrosis and inflammatory response via the miR-181a/Egr-1/TLR4 axis in vitro and in vivo. These results provide new insights into the regulation between MEG3 and the miR-181a/Egr-1/TLR4 signaling pathway during DN progression.

## Introduction

As the main complication of diabetes-related kidney damage, diabetic nephropathy (DN) is a complex clinical problem worldwide [1,2]. The high frequency of DN in diabetics has been observed despite considerable advancements in therapeutic approaches to diabetes [3,4]. An increasing number of research works have been performed to investigate the mechanisms of DN progression. However, finding effective molecular targets for DN clinical treatment is challenging.

Long non-coding RNAs (lncRNAs) are a vital group of non-coding RNA molecules that are >200 nucleotides in length [5,6]. The novel biologic roles of lncRNAs in DN have been confirmed due to the rapid development of RNA genomics. LncRNA ENSMUST00000147869 inhibits fibrosis and proliferation in mesangial cells (MCs) induced by DN [7]. MIAT/Nrf2 is a vital pathway for renal tubular epithelial injury induced by high glucose [8]. MALAT1 is upregulated in DN and is involved in podocyte injury induced by high glucose via β-catenin [9]. Long non-coding RNA maternally expressed gene 3 (lncRNA MEG3, ID: 55384) is a novel lncRNA that acts as a tumor suppressor in multiple cancer cells. For example, MEG3 suppresses invasion and proliferation by promoting the ubiquitination of EZH2 in gallbladder cancer [10]. MEG3 also inhibits growth by suppressing β-catenin in liver cancer cells via the PKM2/PTEN pathway [11]. However, the potential mechanism and biological function of MEG3 in DN are still barely elucidated.

MicroRNAs (miRNAs) are small noncoding RNAs that are 20–25 nucleotides in length [12,13]. MiRNAs regulate the expression of downstream targets via post-transcriptional modulation [14]. LncRNAs can function as competing endogenous RNAs (ceRNAs), which compete for miRNA binding and block miRNA-mediated target gene silencing [15]. The regulating network of ceRNAs is suggested to play important roles in DN development. LncRNA LINC01619 regulates podocyte injury via the miR-27a/FOXO1 pathway in DN [16]. NEAT1 enhances EMT and extracellular matrix accumulation by targeting the miR-27b-3p/ZEB1 pathway in DN [17]. LncRNA-NR_033515 enhances EMT, proliferation, and fibrogenesis by targeting miR-743b-5p in DN [18].

In the current research, we found that MEG3 was upregulated in DN in vivo and in vitro. MEG3 could enhance cell fibrosis and inflammatory response in MCs and could interact with miR-181a. Our study further demonstrated that MEG3 promotes fibrosis and inflammatory response in DN through the miR-181a/Egr-1/TLR4 pathway.

## RESULTS

### MEG3 expression was upregulated in DN in vivo and in vitro

**Figure 1:**
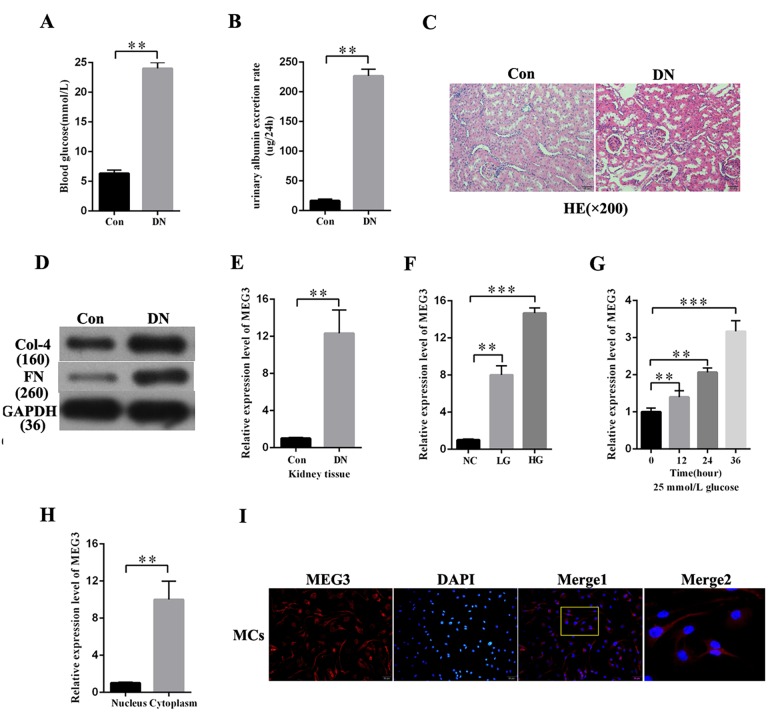
**Expression level of MEG3 was upregulated in DN.** (**A**) Blood glucose level, (**B**) urinary albumin excretion rate, (**C**) representative kidney histology of HE staining, and (**D**) DN markers (Col-4 and FN) in kidney sections in negative control and DN. (**E**) MEG3 expression was upregulated in DN tissues relative to that in the negative control; (**F**) MEG3 expression was measured in MCs stimulated with D-glucose at 5.5 (NC), 19.5 (LG), and 25 mmol/L (HG); (**G**) MEG3 expression was measured in MCs stimulated with 25 mmol/L glucose for 0, 12, 24, and 36 h; (**H**–**I**) qPCR and FISH results showed that MEG3 was mainly expressed in the cytoplasm of MCs.

DN rat models were established to investigate the expression profile of MEG3 in DN. As respectively shown in Figures 1A and B, blood glucose level and urinary albumin excretion rate were significantly increased in DN rat models compared with those in the negative group. HE staining showed morphological glomerular changes in diabetic kidney tissue (Figure 1C). The expression level of fibrosis markers (Col-4 and FN) was increased in DN compared with that in the negative group (Figure 1D). MEG3 expression in DN was detected. QRT-PCR results indicated that MEG3 was upregulated in DN tissues (Figure 1E). MEG3 expression was measured in MCs stimulated by D-glucose at 5.5 (NC), 19.5 (LG), and 25 mmol/L (HG) and was found to increase with glucose concentration (Figure 1F). MEG3 expression in MCs cultured in D-glucose at 25 mmol/L was measured at 0, 12, 24, and 36 h. The results showed that MEG3 expression was upregulated with time (Figure 1G). The lncRNAs must be mainly expressed in the cytoplasm to act as an effective miRNA sponge. The qPCR and fluorescence in situ hybridization (FISH) results revealed that MEG3 was mainly localized in the cytoplasm of MCs (Figures 1H–1F). Thus, these results showed that MEG3 expression was upregulated in DN and mainly localized in the cytoplasm of MCs.

### MEG3 upregulated the expression of fibrosis-related proteins in MCs

**Figure 2:**
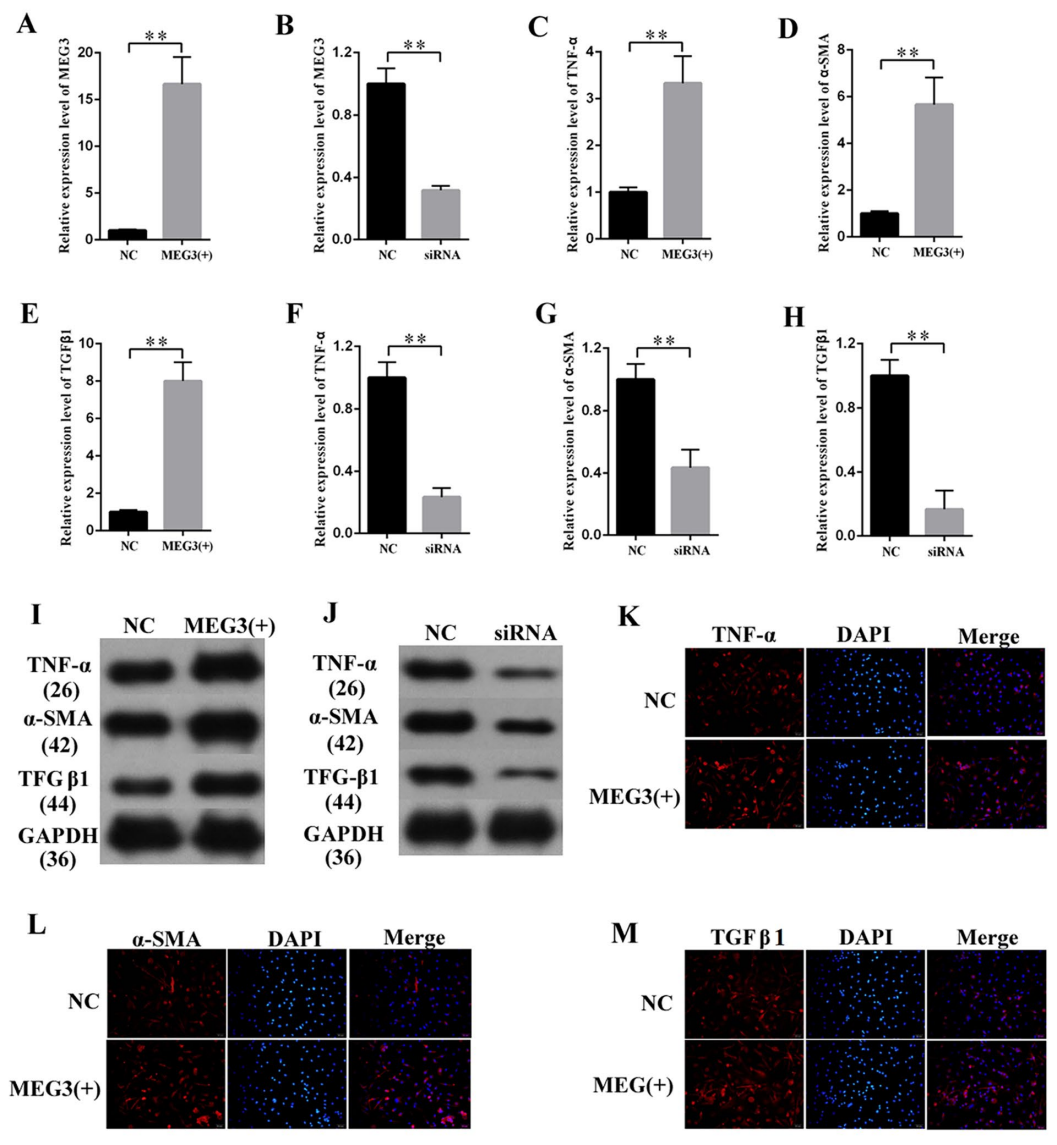
**MEG3 upregulated the expression levels of fibrosis-related proteins in MCs.** (**A**–**B**) The efficiency of LV-MEG3 and siRNA-MEG3 was determined by qRT-PCR; (**C**–**E**) The mRNA expression levels of TNF-α, α-SMA, and TGF-β1 were upregulated in MCs transfected with LV-MEG3; (**F**–**H**) The mRNA expression levels of TNF-α, α-SMA, and TGF-β1 were downregulated in MCs transfected with siRNA-MEG3; (**I**) MEG3 overexpression upregulated the protein expression levels of TNF-α, α-SMA, and TGF-β1 in MCs. (**J**) MEG3 silencing downregulated the protein expression level of TNF-α, α-SMA, and TGF-β1 in MCs. (**K**–**M**) immunofluorescence analysis results revealed the upregulated expression of TNF-α (**K**), α-SMA (**L**), and TGF-β1 (**M**) in MCs transfected with LV-MEG3.

Fibrosis changes are the pathologic bases of DN. MCs are the major participants in the development of fibrosis changes. MEG3 overexpression or knockdown was performed to study the effects of MEG3 on the fibrosis of MCs. The efficiency of lentiviral vector for MEG3 (LV-MEG3) and siRNA-MEG3 was determined by qPCR (Figures 2A–2B, respectively). First, the mRNA expression of fibrosis-related protein was quantified via qPCR (Figures 2C–2H), which revealed that MEG3 overexpression raised the mRNA expression level of TNF-α (Figure 2C), α-SMA (Figure 2D), and TGF-β1 (Figure 2E). MEG3 silencing decreased the mRNA expression of TNF-α (Figure 2F), α-SMA (Figure 2G), and TGFβ1 (Figure 2H). Western blot results showed that overexpression (Figure 2I) and silencing (Figure 2J) of MEG3 respectively enhanced and decreased the protein expression of TNF-α, α-SMA, and TGFβ1. Immunofluorescence analysis was also performed, revealing that MEG3 promoted fibrosis-related proteins in MCs (Figures 2K–2M). These findings demonstrate that MEG3 enhanced cell fibrosis and upregulated the expression of fibrosis-related proteins in MCs.

### MEG3 enhanced the expression of inflammatory cytokines in vitro and in vivo

Increased inflammation is a vital mechanism in the progression of DN. MEG3 overexpression or knockdown was performed to study the effects of MEG3 on the inflammatory response in DN.

**Figure 3:**
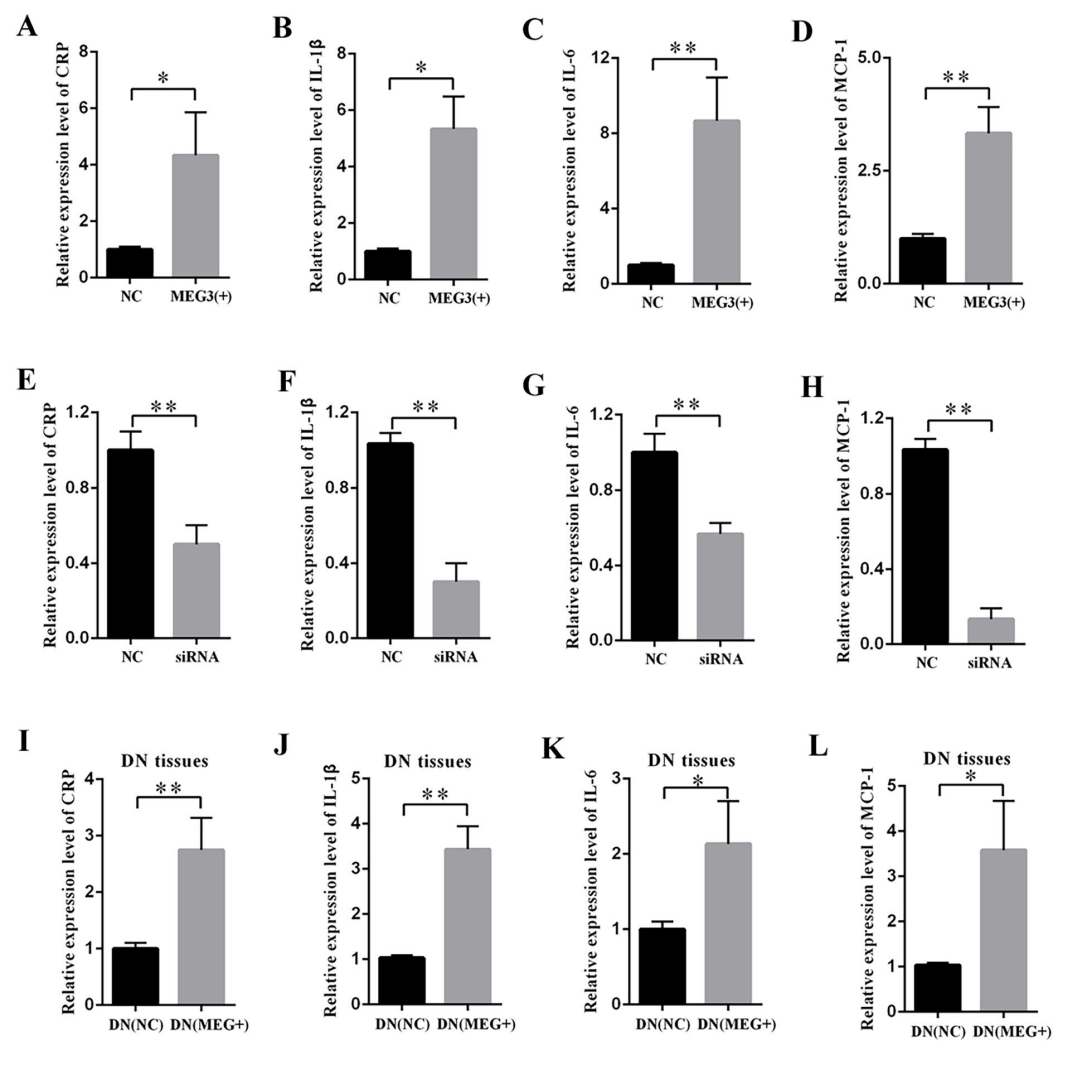
**Effect of MEG3 on the secretion of inflammatory cytokines in vitro and in vivo.** (**A**–**D**) ELISA analysis results showed that the concentrations of CRP (**A**), IL-1β (**B**), IL-6 (**C**), and MCP-1 (**D**) in the supernatant of MCs were upregulated. MCs were transfected with LV-MEG3; (**E**–**H**) ELISA analysis results showed that the concentrations of CRP (**E**), IL-1β (**F**), IL-6 (**G**), and MCP-1 (**H**) in the supernatant of MCs were downregulated. MCs were transfected with siRNA-MEG3; (**I**–**L**) The concentrations of CRP (I), IL-1β (**J**), IL-6 (**K**), and MCP-1(**L**) in the kidney cortex of DN rats were upregulated. DN rats were injected with LV-MEG3.

The supernatant of MCs was collected, and DN-related inflammatory cytokines were measured by ELISA. The results revealed that MEG3 overexpression raised the concentrations of CRP (Figure 3A), IL-1β (Figure 3B), IL-6 (Figure 3C), and MCP-1 (Figure 3D). By contrast, MEG3 silencing decreased the concentrations of CRP (Figure 3E), IL-1β (Figure 3F), IL-6 (Figure 3G), and MCP-1 (Figure 3H). DN rats were injected with LV-MEG3 to detect the effect of MEG3 in vivo. ELISA results indicated that MEG3 overexpression increased CRP (Figure 3I), IL-1β (Figure 3J), IL-6 (Figure 3K), and MCP-1 (Figure 3L) expression in the kidney cortex of DN rats. Therefore, these data indicate that MEG3 overexpression promoted inflammation in DN.

### MEG3 interacted with miR-181a via a direct targeting and Ago2-dependent manner

**Figure 4:**
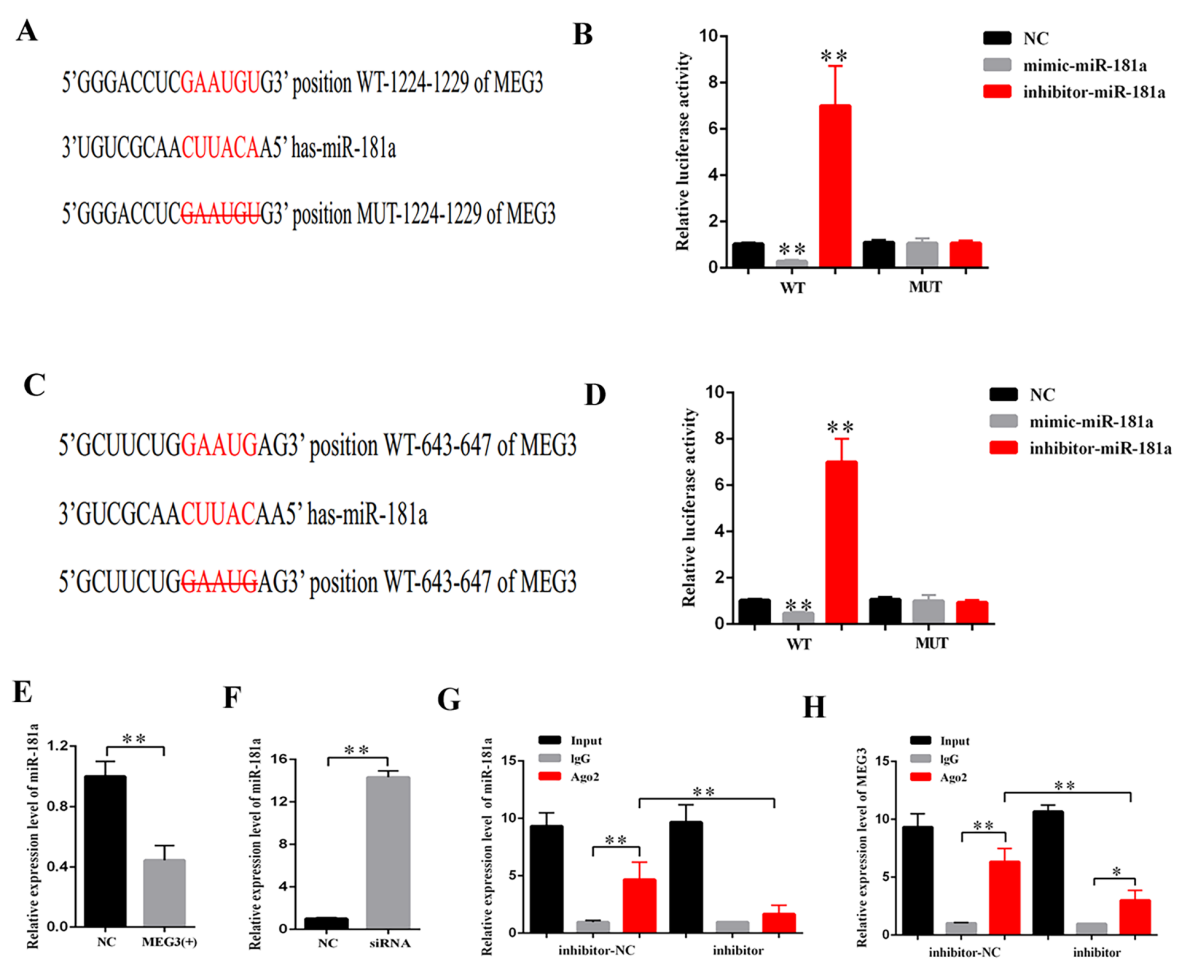
**MEG3 sponging with miR-181a via direct targeting and an Ago2-dependent manner.** (**A**–**D**) Direct binding sites between MEG3 and miR-181a (**A** and **C**). Luciferase reporter assay was performed for the confirmation of direct binding relationship between MEG3 and miR-181a with luciferase reporter plasmids of wild- and mutant-type MEG3 (**B** and **D**). (**E**–**F**) QRT-PCR demonstrated that the overexpression and silencing of MEG3 respectively decreased and increased the expression level of miR-181a; (**G**–**H**) RIP assay was performed using input from cell lysate, IgG, or anti-Ago2. The relative expression levels of MEG3 and miR-181a were detected by qPCR.

CeRNA functions as a vital regulatory mechanism in DN [19,20]. StarBase v2.0 (http://starbase.sysu.edu.cn/index.php) was used to predict the potential target miRNA of MEG3. Some of the results are presented in Supplementary Table 1. MiR-181a plays a vital role in fibrosis and inflammation [21,22]. Thus, miR-181a, which contains two binding sites in MEG3 transcript, was selected as the prediction candidate. Luciferase reporter assay was performed to confirm the direct binding relationship between MEG3 and miR-181a. This binding relationship followed the basic complementary pairing principle. Thus, an MEG3 mutant-type reporter, in which binding sites between MEG3 and miR-181a were knocked out, was built. The results showed that the luciferase activity of MEG3 wild- and mutant-type reporter genes were respectively suppressed and not inhibited by mimic-miR-181a. The luciferase activity of MEG3 wild- and mutant-type reporter genes were respectively increased and decreased by miR-181a inhibitor (Figures 4A–4D). In addition, qPCR results revealed that MEG3 upregulation and downregulation respectively decreased and increased miR-181a expression (Figures 4E–4F). RNA-induced silencing complexes (RISCs) play a central role in posttranscriptional gene regulation. LncRNAs are a vital part of RISCs. MiRNAs bind their targets to repress and/or degrade RNA through RISC in an Ago2-dependent manner [23,24]. In the current study, RNA immunoprecipitation (RIP) assay results showed that MEG3 and miR-181a expression were increased in the anti-Ago2 group compared with that in the control group. The expression levels of MEG3 and miR-181a immunoprecipitated with Ago2 in anti-miR-181a were considerably decreased compared with those in the anti-normal IgG group (Figures 4G–4H). Thus, MEG3 interacted with miR-181a by a direct targeting and Ago2-dependent manner.

### MiR-181a inhibition promoted fibrosis and inflammatory response in MCs

**Figure 5:**
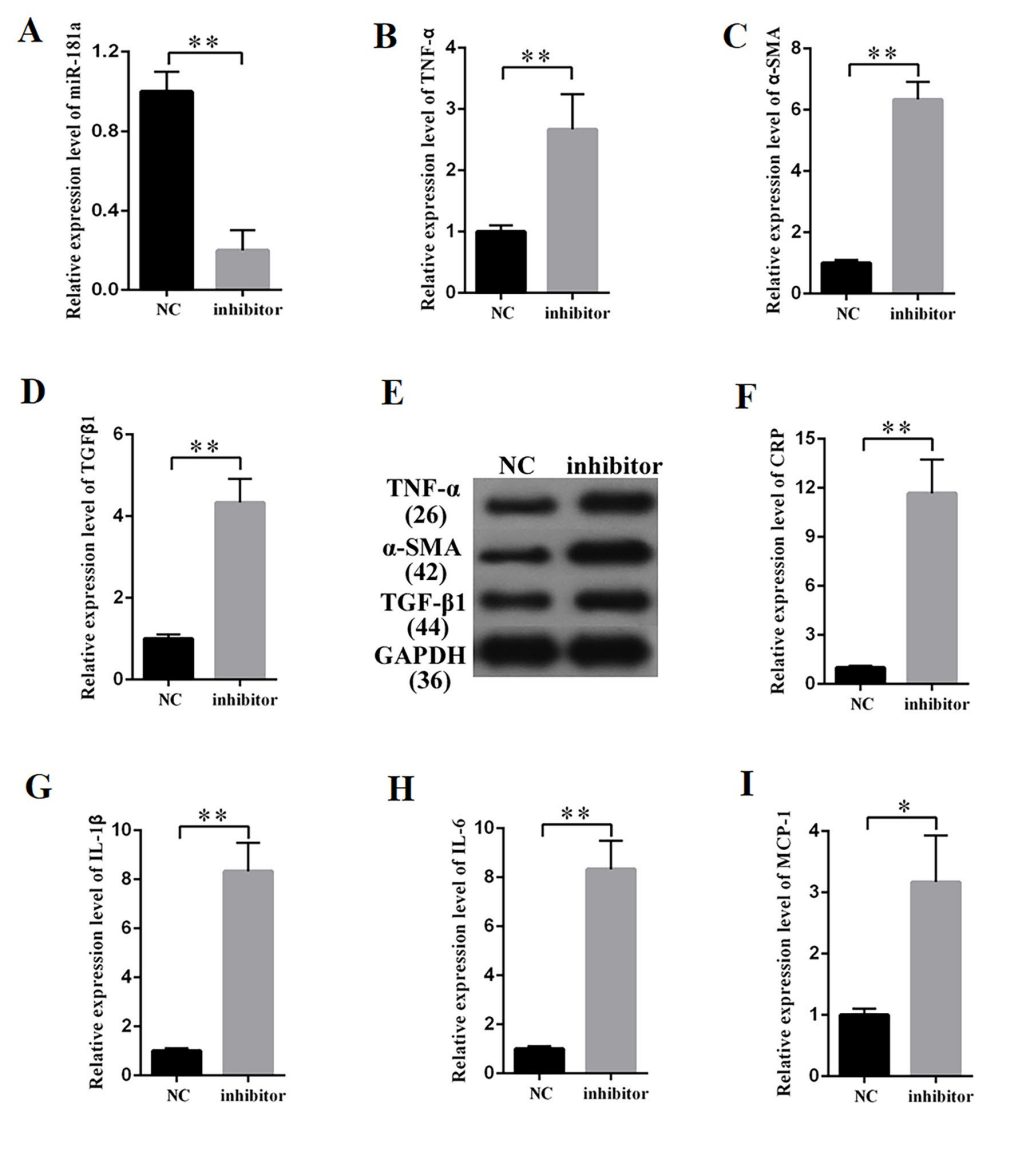
**Inhibition of miR-181a upregulated the expression of fibrosis-related proteins and concentration of inflammatory cytokines.** (**A**) The efficiency of miR-181a inhibitor was determined by qPCR. (**B**-**D**) MiR-181a inhibition increased the mRNA expression level of TNF-α (**B**), α-SMA (**C**), and TGF-β1 (**D**) in MCs based on ELISA. (**E**) MiR-181a inhibition increased the protein expression level of TNF-α, α-SMA, and TGF-β1 in MCs. (**F**-**I**) MiR-181a inhibition increased the concentrations of CRP (**F**), IL-1β (**G**), IL-6 (**H**), and MCP-1 (**I**) in MCs based on ELISA.

MiR-181a was knocked down to study the effects of miR-181a on the fibrosis and inflammatory response in MCs. The efficiency of a miR-181a inhibitor was determined by qPCR (Figure 5A), which revealed that miR-181a inhibition enhanced the mRNA expression of TNF-α, α-SMA, and TGF-β1 in MCs (Figures 5B, 5C, and 5D). Western blot results revealed that miR-181a inhibition increased TNF-α, α-SMA, and TGF-β1 protein expression in MCs (Figure 5E). Subsequently, the supernatant of MCs transfected with miR-181a was collected and subjected to the measurement of inflammatory cytokines by ELISA. ELISA analysis results revealed that miR-181a inhibition increased the concentrations of CRP, IL-1β, IL-6, and MCP-1 in MCs (Figures 5F, 5G, 5H, and 5I). In summary, miR-181a inhibition enhanced the expression of fibrosis-related proteins and the concentration of inflammatory cytokines in MCs.

### MiR-181a increased the expression of fibrosis-related proteins and concentrations of inflammatory cytokines through Egr-1/TLR4 signaling

**Figure 6:**
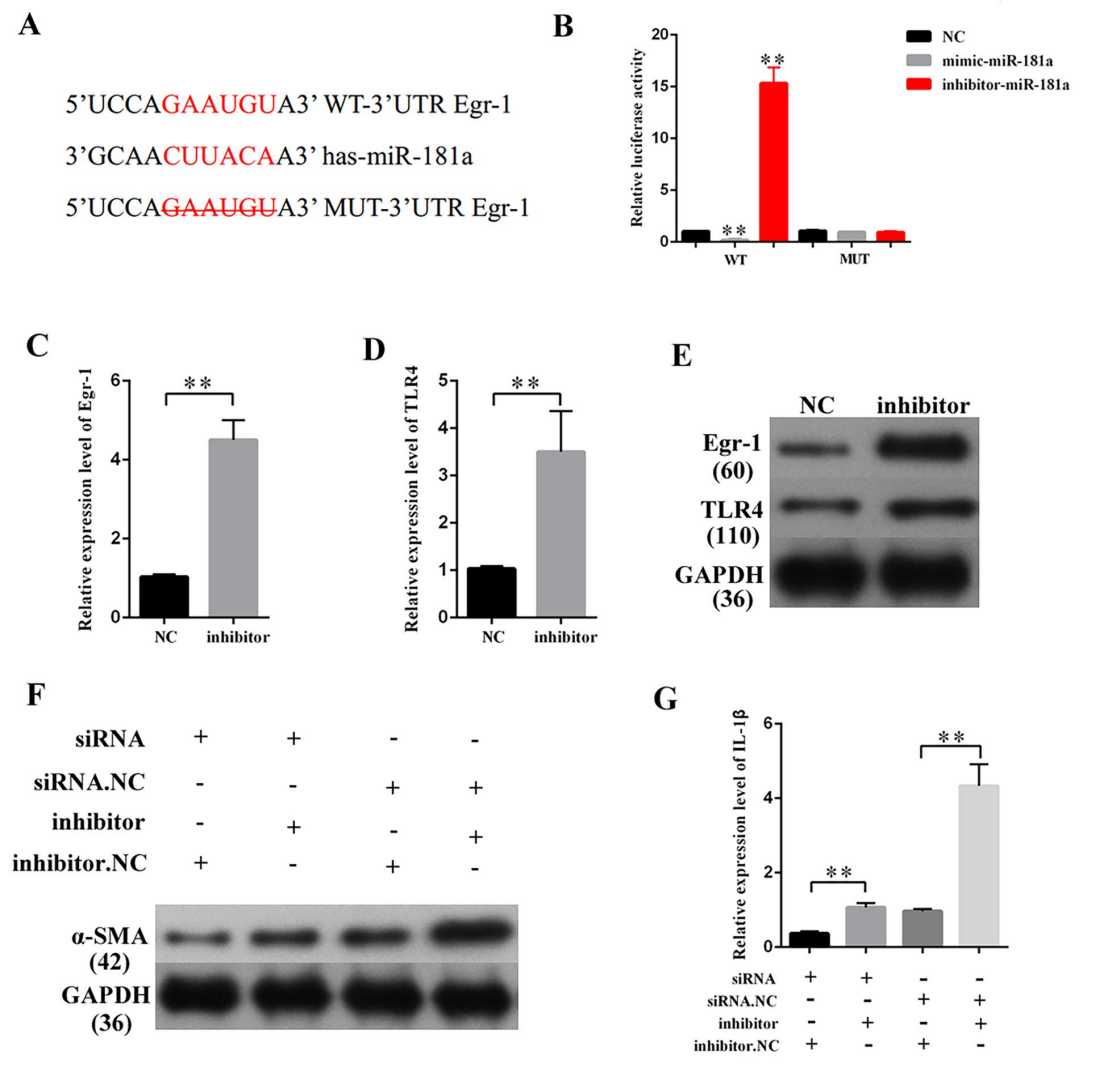
**MiR-181a increased the expression of fibrosis-related proteins and concentration of inflammatory cytokines through Egr1/TLR4 signaling.** (**A**) Binding region between miR-181a and Egr1. (**B**) Luciferase reporter assay for the confirmation of direct binding relationship between miR-181a and Egr1 was performed with luciferase reporter plasmids of wild- or mutant-type Egr1; (**C**) Egr-1 mRNA expression was upregulated in MCs transfected with miR-181a inhibitor. (**D**) TLR4 mRNA expression was upregulated in MCs transfected with miR-181a inhibitor. (**E**) miR-181a inhibition increased the protein level of Egr-1 and TLR4. (**F**) The protein expression of α-SMA was measured in MCs by Western blot. Cells were transfected with inhibitor-miR-181a or pcDNA3.1-Egr1. (**G**) The concentration of IL-1β in MCs was measured by ELISA. The cells were transfected with miR-181a inhibitor or pcDNA3.1-Egr1.

TargetScan (http://www.targetscan.org/vert_72/) was used to predict the potential target gene of miR-181a. Some of the results are presented in Supplementary Table 2. Egr-1 participates in the development of DN [25]. Thus, Egr-1, which contains one binding site in miR-181a transcript (Figure 6A), was selected for miR-181a candidate prediction. The binding relationship between miR-181a and Egr-1 follows the basic complementary pairing principle. If the binding sites are knocked out, then miRNA cannot bind the 3′UTR of the target gene. Thus, an Egr-1 mutant-type reporter, in which binding sites between miR-181a and Egr-1 are knocked out, was built. Luciferase reporter assay results showed that the luciferase activity of Egr-1 wild-and mutant-type reporter genes were and were not suppressed by mimic-miR-181a and miR-181a inhibitor, respectively. The luciferase activities of Egr-1 wild- and mutant-type reporter genes were respectively increased and decreased by the miR-181a inhibitor (Figure 6B). TLR4 is a gene located downstream of Egr-1 [26]. QRT-PCR and Western blot results showed that miR-181a inhibition increased the expression of Egr-1 and TLR4 (Figures 6C–6E). Rescuing experiment results showed that Egr-1 knockdown restored the marked upregulation of α-SMA in MCs transfected with miR-181a inhibitor. Moreover, ELISA indicated that Egr-1 knockdown restored the marked upregulation of IL-1β in MCs transfected with miR-181a inhibitor. Thus, miR-181a inhibition upregulated the expression levels of fibrosis-related proteins and concentrations of inflammatory cytokines through the Egr-1/TLR4 axis.

### MEG3 promoted Egr-1 expression, fibrosis, and inflammatory response by acting as a miR-181a sponge

**Figure 7:**
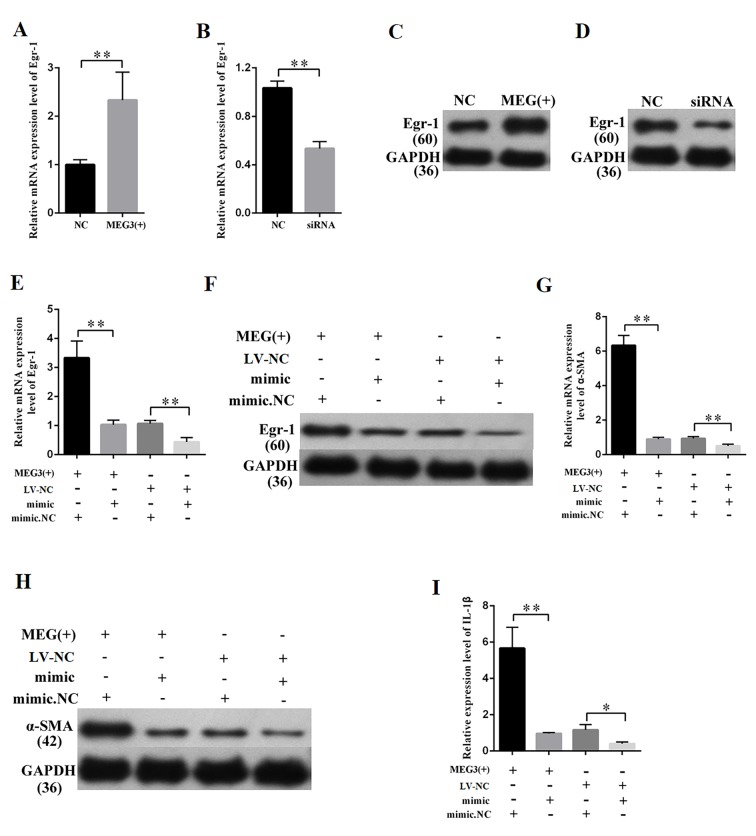
**MEG3 promoted Egr1 expression and enhanced the fibrosis and inflammatory response of MCs by acting as a miR-181a sponge.** (**A**, **B**) QPCR results showed that the expression level of Egr-1 was increased and decreased in MCs transfected with LV-MEG3 and siRNA-MEG3, respectively. (**C**, **D**) Western blot results showed that overexpression and knockout of MEG3 increased and decreased the expression level of Egr-1, respectively. (**E**, **F**) QPCR and Western blot revealing Egr-1 expression in MCs. The cells were transfected with mimic-miR-181a or LV-MEG3. (**G**, **H**) QPCR and Western blot showed the expression level of α-SMA in MCs. The cells were transfected with mimic-miR-181a or LV-MEG3. (**I**) The concentration of IL-1β in MCs was measured by ELISA. The cells were transfected with mimic-miR-181a or LV-MEG3.

MEG3 overexpression and knockdown were performed to further study the relationship between MEG3 and Egr-1. QRT-PCR and Western blot results revealed that the overexpression and silencing of MEG3 respectively increased and decreased the Egr-1 expression (Figures 7A–7D). QRT-PCR and Western blot were then performed by respectively co-transfecting LV-MEG3 and mimic-miR-181a into MCs to investigate whether miR-181a mediates the function of MEG3. The results showed that mimic-miR-181a restored Egr-1 upregulation by MEG3 overexpression (Figures 7E–7F). QRT-PCR and Western blot indicated that miR-181a overexpression attenuated the promotive roles of MEG3 on α-SMA (Figures 7G–7H). ELISA indicated that miR-181a overexpression attenuated the promotive roles of MEG3 on IL-1β (Figure 7I). In summary, MEG3 upregulated Egr-1 expression and enhanced fibrosis and inflammatory response by acting as a miR-181a sponge.

### MEG3 overexpression enhanced DN development in vivo

**Figure 8:**
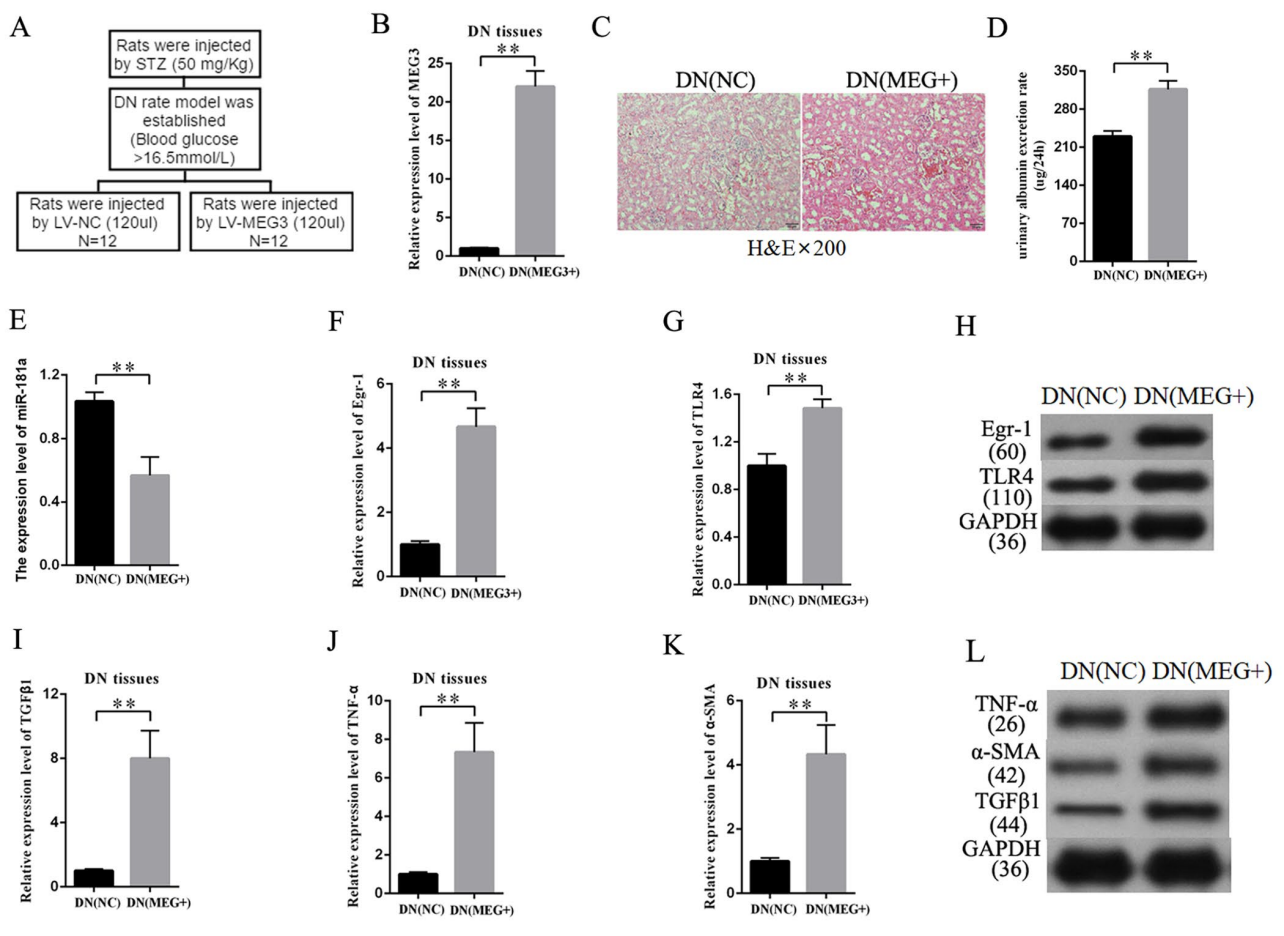
**MEG3 overexpression enhanced DN development in vivo.** (**A**) Establishment procedure of overexpressed MEG3 in DN rat models (LV-MEG3 group = 12, LV-NC group = 12). (**B**) QPCR results showed that MEG3 was successfully overexpressed in DN tissues after LV-MEG3 injection. (**C**) HE staining results as demonstrated in the microscope. (**D**) MEG3 overexpression increased the urinary albumin excretion rate in DN rat models. (**E**) QPCR results showed that miR-181a expression was downregulated in DN after LV-MEG3 injection. (**F**, **G**) MEG3 overexpression increased the mRNA expression of Egr-1 and TLR4. (**H**) MEG3 overexpression increased the protein expression of Egr-1 and TLR4 in vivo. (**I**–**K**) MEG3 overexpression increased the mRNA expression of TGFβ1 (**H**), TNF-α (**I**), and α-SMA (**J**) in vivo. (**L**) Western blot results indicated that MEG3 overexpression increased the protein expression of TGFβ1, TNF-α, and α-SMA.

An overexpression-MEG3 DN rat model was built to further study the effects of MEG3 on DN in vivo. The establishment procedure is shown in Figure 8A. The efficiency of LV-MEG3 was detected in kidney tissues by qRT-PCR. The results indicated that MEG3 was successfully overexpressed in DN tissues after LV-MEG3 injection (Figure 8B). H&E staining results showed that MEG3 overexpression promoted DN development (Figure 8C) and increased urinary albumin excretion rate in DN rat models compared with that in the NC group (Figure 8D). The expression of miR-181a, Egr-1, and TLR4 was detected in kidney tissues to validate the participation of the miR-181a/Egr-1/TLR4 axis in the roles of MEG3 in DN. MEG3 overexpression decreased miR-181a expression (Figure 8E). QRT-PCR results indicated that Egr-1 and TLR4 expression was enhanced in DN tissues by LV-MEG3 injection (Figures 8F–8G). Western blot results indicated that MEG3 overexpression upregulated Egr-1 and TLR4 protein expression in DN tissues (Figure 8H). In addition, fibrosis-related genes were detected in the DN rat model. QPCR and Western blot results showed that TNF-α, α-SMA, and TGF-β1 were upregulated in the overexpression-MEG3 DN rat model (Figures 8I–8L). These results demonstrated that MEG3 overexpression enhanced DN development via the miR-181a/Egr-1/TLR4 axis.

## DISCUSSION

DN is a common cause of renal failure [27]. Medical treatment for this disease is currently unavailable [28]. Numerous patients with DN require kidney dialysis throughout life. Thus, understanding the potential molecular mechanism of DN is important for the discovery of novel therapeutic targets and the development of new specific drugs.

CeRNA has become an important mechanism of lncRNAs to participate in the regulation of coding gene expression in DN. LncRNA NR_033515 enhances EMT and fibrogenesis by acting as a sponge for miR-743b-5p in DN [18]. LncRNA 1700020I14Rik inhibits cell fibrosis and proliferation in DN by targeting miR-34a-5p [29]. LncRNA Erbb4-IR enhances DN-related injury by targeting miR-29b [30]. MEG3 plays a vital role in diabetes [31]. Genetic variants of MEG3 confer risk of type 2 diabetes in populations [32]. MEG3 enhances hepatic insulin resistance via miR-214/ATF4 axis [33]. However, the biological role and specific underlying mechanisms of MEG3 in DN are completely unknown.

In this research, we found that MEG3 expression level was remarkably enhanced in DN in vivo and vitro. QPCR and FISH results showed that MEG3 was mainly localized in the cytoplasm of MCs. MEG3 enhanced fibrosis and inflammatory response in MCs. MEG3 overexpression improved the expression of fibrosis-related proteins and inflammatory cytokines. Conversely, MEG3 silencing suppressed fibrosis and inflammatory response. MEG3 is mainly located in the cell cytoplasm, thereby suggesting that MEG3 also plays a role in MCs by ceRNA. Bioinformatics analysis predicted MEG3 putative binding sites with miR-181a. Luciferase assay and RIP assay showed that MEG3 interacts with miR-181a through direct targeting by and in an Ago2-dependent manner, respectively. MiR-181a inhibition suppressed fibrosis and inflammatory response in MCs. Egr-1 was demonstrated to be a direct target gene of miR-181a. Egr-1 is involved in the development of DN. Egr-1 silencing inhibits renal tubulointerstitial fibrosis in DN [25]. Egr-1 also increases pro-fibrotic gene expression in a high-sugar environment in HK-2 cells [34]. In the current study, a rescuing experiment revealed that MEG3 promoted Egr-1 expression, fibrosis, and inflammatory response in a ceRNA-dependent manner. Finally, MEG3 could enhance the development of DN in vivo. In DN rat models, MEG3 overexpression increased the urinary albumin excretion rate and upregulated the expression of fibrosis proteins. MEG3 upregulation decreased miR-181 expression and increased Egr-1 and TLR4 expression

**Figure 9:**
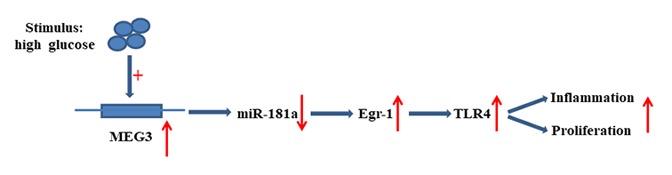
Relationship between MEG3, miR-181a, Egr1, and TLR4.

The present research revealed that MEG3 functions as ceRNA to upregulate Egr-1 expression by acting as a sponge for miR-181a. MEG3 promoted fibrosis and inflammatory response in DN. The relationship between MEG3, miR-181a, Egr1, and TLR4 is illustrated in Figure 9. Therefore, MEG3 may act as a potential therapeutic target and biomarker for further DN clinical application.

## MATERIALS AND METHODS

### Cell culture

Cell culture was performed in accordance with a previous study [35]. RAW264.7 cells were cultured in RPMI1640 (Gibco, USA) supplemented with 20% FBS (Gibco, USA) in a 37 °C incubator with 5% CO_2_. MCs were cultured in 5.5 mmol/L glucose, low glucose (LG, 5.5 mmol/L glucose plus 19.5 mmol/L mannitol), or high glucose (HG, 25 mmol/L glucose).

### Cell transfection, lentivirus production, and transduction

A lentiviral vector for MEG3 was constructed by Bio-Link Gene (China). MiR-181a mimic, miR-181a inhibitor, and the corresponding controls were purchased from RiboBio (Guangzhou, China). The sequence of Egr-1 was cloned into a pcDNA3.1 vector. An empty vector functioned as negative control. Lipofectamine 3000 (Invitrogen, USA) was used for transfection in accordance with the manufacturer’s instructions.

### ELISA

ELISA was performed in accordance with a previous study [36]. The supernatants of MCs and DN tissues in lysis buffer were collected and measured by CRP, IL-1β, IL-6, and MCP-1 ELISA Kits (Boster Bioengineering Co, China) in accordance with the manufacturer’s instructions.

### Isolation of nuclear and cytoplasmic RNA

Nuclear and cytoplasmic RNA were isolated in accordance with a previous work [16]. Cytoplasmic and nuclear RNA were extracted and separated using a Nuclear and Cytoplasmic RNA Purification Kit (Norgen, USA). The isolated RNA was detected by qRT-PCR.

### Fluorescence in situ hybridization

MCs were permeabilized with pre-cooled Triton X-100 (0.1%), and the slides were treated with 4% paraformaldehyde. Hybridization was then performed overnight with the lncRNA MEG3 probe. The cells were washed with SCC buffer. DAPI was used to stain the coverslip, and laser scanning confocal microscopy was utilized to test fluorescence.

### QRT-PCR

All RNAs were isolated using the TRIzol reagent (Life Technologies, US). MRNA expression was measured via SYBR Green qPCR on an ABI 7300 real-time PCR machine. TNF-α, α-SMA, and TGF-β1 mRNA expression levels were respectively examined using the following specific primers: F-5′-TTGTCCTCAGTTTCGGGAGAT-3′ R-5′-CAAGTCTGTGTCCTGAACGC-3′; F-5′-CCTGTGTTGTGGTTTACACTGG-3′ R-5′- GGGGGAATTATCTTTCCTGGT; F-5′-AGGAGCAAAATCTGTCCGATCT-3′ R-5′- GTGGGGGAATTATCTTTCCT.

### Bioinformatics analysis

The candidate target miRNA of MEG3 was analyzed using StarBase v2.0 (http://starbase.sysu.edu.cn/index.php). The candidate target genes of miR-181a were analyzed via the miRNA target prediction program miRNA.org (http://www.microrna.org/microrna/home.do).

### Western blot

Protein (16–20 mg) extracted from the samples was utilized for Western blot. Antibodies utilized in the research included anti-TNF-α (1:1000; HK), anti-α-SMA (1:1000; HK), anti-TGF-β1 (1:1000; HK), anti-Egr-1 (1:1000; HK), and anti-TLR4 (1:1000; HK), and GAPDH (1:1000; USA) was utilized as loading control.

### Luciferase reporter assay

Luciferase reporter assay was performed according to a previous study [37]. Specifically, pmirGLO-MEG-WT or pmirGLO-Egr-1-WT reporter plasmids (120 ng) and corresponding mutated vectors were transfected into 293T cells with miR-181a inhibitor or miR181a mimic (100 nM) with Lipofectamine 3000. In accordance with standard protocols, 293T cells were lysed 48 h after transfection, and luciferase activity was measured using the PicaGene Dual SeaPansy luminescence kit (Toyo Ink, Japan).

### RIP assays

RIP assay was performed according to the literature [35] to determine the association between 1700020I14Rik and the RISC. The cell lysate was incubated with RIP immunoprecipitation buffer, which contained magnetic beads conjugated with Ago2 antibody (Abcam, USA) and NC IgG (Abcam, Cambridge, USA). RIP experiments were performed using Magna RIP kit (Millipore, USA). Co-precipitated RNAs were detected via qRT-PCR.

### Immunofluorescence assay

For immunofluorescence analysis, the fixed cells were permeabilized with 0.1% Triton X-100 and blocked in 5% goat serum solution. The coverslips were added with primary antibodies against TNF-α (HK, 1:50), α-SMA (HK, 1:50), or TGF-β1 (HK, 1:50) at 4 °C overnight and then with Cy3-labeled secondary antibody (Proteintech, USA, 1:50) for 1 h at room temperature. After treatment with DAPI, the coverslips were sealed with glycerine. Images were observed with QImaging Micro-publisherTM 5.0 RTV (Olympus Corporation, Japan) and analyzed with Image-Pro Plus (Medium Cybernetics, Bethesda, MD, USA).

### In vivo study

Eight-week-old male rats (SD rats) were purchased from Shanghai Laboratory Animal Center (Shanghai, China) and kept in an SPF lab. Two groups were set up (*N* = 12 for each group): DN + LV‐NC group and DN + LV‐MEG3 group. Streptozotocin (STZ) was used to establish the DN rat model. STZ was dissolved in 0.1 M citrate acid buffer, which was used to build DN rat models (50 mg/kg). The rats were injected once daily for six days. Blood glucose levels of >16.5 mmol/L were identified as successful DN models. Lentiviral –MEG3 (LV-MEG3) was used to establish the DN rat model. LV‐MEG3 or LV‐NC (120 µl) was injected into DN models. All DN rat models were sacrificed at the end of the third week after injection. The study was approved by the Ethics Committee of Qingpu Branch of Zhongshan Hospital Affiliated to Fudan University, and the procedures were performed in accordance with the regulations of the Ethics Committee.

### Statistical analysis

Student’s t-test and one-way ANOVA were performed for statistical analysis by using the GraphPad Prism 5.0. Statistical data are expressed as mean ± standard deviation (SD). Diﬀerences were considered significant at P < 0.05.

## Supplementary Material

Supplementary Tables
