# Supplementary material for: Long non-coding RNA MEG3 promotes fibrosis and inflammatory response in diabetic nephropathy via miR-181a/Egr-1/TLR4 axis
Source: Aging (Albany NY). 2019 Jun 13;11(11):3716–30. doi: 10.18632/aging.102011 (PMC6594792; doi:10.18632/aging.102011)
Supplement: Supplementary Tables [file aging-11-102011-s001.pdf]

SUPPLEMENTARY TABLES

Supplementary Table 1. A part predicted target miRNAs of MEG3.

miRNA	Alignment
miR-542-3p	Target: 5' uggUAGUGAAUGUUUCUGUCACu 3'<br>miRNA:3' aaaGUCA-AUAGUUAGACAGUGu 5
miR-181a	Target:5'GGGACCUCGAAUGUG3'<br>miRNA:3'UGUCGCAACUUACAA5'<br>Target:5'GCUUCUGGAAUGAG3'<br>miRNA:3'GUCGCAACUUACAA5'
miR-1245b-5p	Target: 5' gccAAGCUUCUUGAAAGGCCUg 3'<br>miRNA : 3' aaaUUC-ACUAGAUUUCCGGAu 5'
miR-320b	Target: 5' ugaaagUUGCAG-GCAGCUUUg 3'<br>miRNA : 3' aacgggAGAGUUGGGUCGAAAa 5'
miR-424-5p	Target: 5' aaCACACAUGUGGCCUUGCUGCUg 3'<br>miRNA : 3' aaGU-UUUGUACU-UAACGACGAc 5'
miR-15b-5p	Target: 5' agaACACACAUGUGGCCUUGCUGCUg 3'<br>miRNA : 3' acaUUUG-GUACUAC---ACGACGAu 5
miR-6766-3p	Target: 5' auuuucUGGGGCACCGACAAUCu 3'<br>miRNA : 3' acucccACCCCCU-UCUGUUAGu 5'

Supplementary Table 2. A part of predicted target genes of miR-181a.

NAP1L5	Tatget:5'GUUUUUCCCAGAAUAUCGAUGGA3'<br>miRNA:3' CCAUGUUAGUUGCCAGCUACCA5'
Egr-1	Target:5'UCCAGAAUGUA3' WT-3'UTR<br>miRNA:3'GCAACUUACAA3'
MAP3K5	Target:5' UGCUCAAUCUAAUCUUCGAUGGA3'<br>miRNA:3' CCAUGUUAGUUGCC-AGCUACCA5'
AGPAT4	Target:5' GGCCUCCGUGGGAGUUCGAUGGA3'<br>miRNA:3'CCAUGUUAGUUGCCAGCUACCA5'
TUBB6	Target:5' AUGAGGAAGAGGAGAUCGAUGGA3'<br>miRNA:3' CCAUGUUAGUUGCCAGCUACCA5'
SMUG1	Target:5' GGUGAUCAAUGAUGAUCGAUGGA3'<br>miRNA:3' CCAUGUUAGUUGCC---<br>AGCUACCA5'
MFSD7	Target:5' AGGUUGCCCGCCACAUCGAUGGA3'<br>miRNA:3' CCAUGUUAGUUGCCAGCUACCA5'
MKNK2	Target:5' AAAAAAAACAACACAUCGAUGGA3'<br>miRNA:3' CCAUGUUAGUUGCC-AGCUACCA5'
